# Cytotoxic FCER1G^+^ innate-like T cells: new potential for tumour immunotherapy

**DOI:** 10.1038/s41392-022-01061-4

**Published:** 2022-06-29

**Authors:** Emma Morrish, Jürgen Ruland

**Affiliations:** 1 Institute of Clinical Chemistry and Pathobiochemistry, School of Medicine, Technical University of Munich, Munich, Germany; 2 TranslaTUM, Center for Translational Cancer Research, Technical University of Munich, Munich, Germany; 3 German Cancer Consortium (DKTK), Heidelberg, Germany; 4 German Center for Infection Research (DZIF), Partner Site Munich, Munich, Germany

**Keywords:** Tumour immunology, Lymphocytes

In a recent study published in *Nature*, Chou and colleagues define a new evolutionarily conserved class of tumour-elicited immune response mediated by a distinct population of T cell receptor (TCR)-positive FCER1G-expressing innate-like T cells with high cytotoxic potential (αβILTCKs).^1^

Targeted immunotherapies and most prominently immune checkpoint blockade (ICB) therapies, brought clinical benefits to tumour patients that were inconceivable 15 years ago.^2^ These ICBs target inhibitory receptors such as PD-1 on tumour infiltrating CD8^+^ cytotoxic T lymphocytes (CTLs) that can recognise mutated cancer cell antigens and thereby enable tumour cell killing. Yet, a significant cohort of cancer patients are non-responsive to ICB treatment and therefore, there is a strong need to discover additional anti-cancer immunomechanisms. Recent work in *Nature* by Chou and colleagues identifies a population of αβILTCKs that exhibit reactivity to unmutated tumour antigens.^1^

**Fig. 1:**
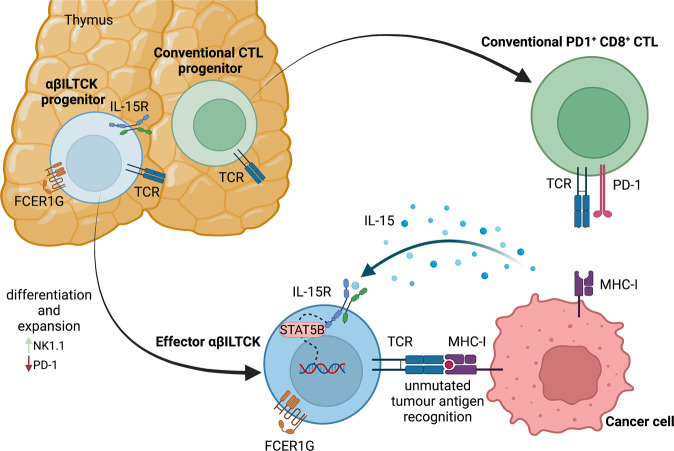
Activation of thymus derived cytotoxic FCER1G^+^ αβILTCKs in cancer tissue. FCER1G-expressing αβ TCR lineage innate-like T cells with high cytotoxic potential (αβILTCKs) are an evolutionarily conserved innate-type T cell that develops from a unique thymic progenitor. These FCER1G^+^ αβILTCKs are distinct from conventional PD-1^+^CD8^+^ cytotoxic T cells (CTLs) and reactive to unmutated self (tumour) antigen. Their intratumoural recruitment, local expansion and effector function is driven by tumour-elicited IL-15 production. For details see text. IL-15R IL-15 receptor, FCER1G Fc Epsilon Receptor Ig, TCR T cell receptor, MHC-I major histocompatibility complex class I. Created with Biorender.com

To comprehensively characterise the phenotypes of tumour-infiltrating cytotoxic T cells, Chou and colleagues started with single cell RNA sequencing analysis of CD45^+^TCRβ^+^CD8α^+^ cells directly isolated from the breast cancer tissues of MMTV-PyMT (PyMT) mice.^1^ Bioinformatic clustering identified five distinct T cell populations including naïve/recently activated, exhausted and proliferative cells, as well as αβILTCKs characterised by high NK1.1 expression.^3^ These cells are characterised by a unique transcriptome that is distinct from that of conventional tumour-infiltrating CD8^+^ T cells. They belong to the unconventional type-1-like innate lymphoid cells and previous work by Dadi and colleagues had shown that αβILTCKs can in principle, exhibit innate cytotoxicity towards tumour cells.^3^ Chou and colleagues also detected the αβILTCKs in murine prostate cancer and human colorectal cancer tissues, indicating that they might represent evolutionarily conserved regulators of tumour-elicited immunosurveillance (Fig. 1).

Because the reactivity, ontogeny and cancer cell sensing mechanisms of αβILTCKs remained unknown,^3^ Chou and colleagues next compared paired-TCR sequences from tumour-resident NK1.1^+^CD8α^+^ αβILTCKs and conventional PD-1^+^CD8α^+^ T cells (PD-1^+^ T cells). From these analyses they could unequivocally conclude that these two tumour infiltrating T cell subsets do not originate from a shared progenitor cell, but represent two mutually exclusive cell fate choices.

As indicated above, immunotherapies that target the PD-1 axis require the activity of conventional PD-1^+^ T cells. These conventional CTLs typically recognise tumours with a high tumour mutational burden due to the ability of their TCRs to detected mutated neoantigens as “non-self”.^4^ In contrast, elegant TCR reporter assays demonstrated that the majority of the TCRs from NK1.1^+^CD8α^+^ αβILTCKs showed reactivity against unmuted antigens from heterologous cancer cell populations.^1^ In this setting, the αβILTCK derived TCRs recognise the cancer-specific peptide in the context of major histocompatibility complex class I (MHC-I) complexes.

To define the developmental origin and dynamics of the tumour infiltrating αβILTCKs, the authors utilised tumour bearing *Fgd5-creER-Rosa26*^*LSL-tdTomato*^ PyMT mice, in which a pulse of tamoxifen injection stably labels Lin^−^c-KIT^+^SCA1^+^ haematopoietic stem cells to allow fate-mapping of their progenitors. The intratumoural αβILTCKs were fate-mapped which demonstrated that this population is replenished by thymic progenitors and in situ proliferation as their primary means of population.

To gain insight into the specifications of the αβILTCK lineage, Chou and colleagues next compared the gene expression profiles of the tumour infiltrating αβILTCKs to that of all other CD45^+^TCRβ^+^CD8α^+^ cells from breast tumour tissues of PyMT mice. Interestingly, expression of *Fcer1g* which encodes for the Fc Epsilon Receptor Ig protein (FCER1G), was differentially expressed within the αβILTCK cluster compared to other tumour infiltrating CD8^+^ T cells, including PD-1^+^ T cells, regardless of their activation status. In primary human colon cancer tissue, FCER1G expression was also detected in TCRβ^+^ cells with a co-receptor expression profile similar to the mouse αβILTCK counterparts. Moreover, these FCER1G^+^ T cells were enriched in cancerous compared to adjacent normal tissue,^1^ indicating that FCER1G is a lineage-defining marker that characterises tumour infiltrating T cells committed to the αβILTCK lineage. Together, these data suggest that the αβILTCK programme in the tumour tissue appears to represent an evolutionarily conserved immune response in both mouse and human.

The immunosurveillance programme of αβILTCKs is critically dependent on the pro-inflammatory cytokine IL-15.^3^ However, the origin of IL-15 that drives the expansion and activation of intratumoural αβILTCKs was previously unknown. Therefore, Chou and colleagues next generated a conditional transgenic breast cancer mouse model in which IL-15 production was specifically ablated within the transformed tumour cells, but not in healthy tissue. Intriguingly, in these *S100a8-cre-Il15*^*fl/fl*^PyMT mice, the loss of cancer-cell derived IL-15 resulted in reduced αβILTCK recruitment into the tumour tissue and impaired cancer immunosurveillance, with accelerated tumour growth in comparison to wild-type controls. Next, the authors constitutively activated IL-15 receptor (IL-15R) signalling by genetically expressing a gain of function STAT5B variant (STAT5B-CA) selectively in αβILTCK progenitors. Adoptive transfer of these engineered αβILTCKs into lymphocyte-deficient tumour-bearing PyMT mice, strongly suppressed tumour growth compared to control αβILTCKs. Even upon transfer into lymphocyte-replete PyMT hosts, these STAT5B-CA expressing αβILTCK progenitors colonised tumour tissue, expanded, differentiated into αβILTCK effector cells, and diminished tumour growth.

In summary, Chou and colleagues identified FCER1G expressing αβILTCKs as a unique and evolutionarily conserved tumour infiltrating cytotoxic T cell population that mediates tumour immune surveillance. Importantly, these cells can be engineered ex vivo and manipulated in vivo to enhance their activity via engagement of the IL-15 signalling pathway. As such, they represent interesting targets for novel tumour immunotherapies. These αβILTCKs can recognise tumour cells with a low mutational burden and their mode of action is distinct from that of conventional PD-1^+^CD8^+^ CTLs. Therefore, utilising these αβILTCKs could be particularly useful against tumours that are refractory to current checkpoint inhibitor therapies. Nevertheless, since the majority of these experiments were completed in murine models, future investigations will need to validate the applicability of αβILTCK-based adoptive cellular therapy in a human model. Moreover, as αβILTCKs recognise still undefined non-mutated antigens which might also be expressed on normal tissue cells, additional studies are required to define potential autoimmune side effects and related toxicities of engineered or IL-15 triggered overactivated αβILTCKs.
